# Case Report: A case of mediastinal parathyroid adenoma leading to hypercalcemic crisis complicated by acute kidney injury and postoperative hungry bone syndrome

**DOI:** 10.3389/fendo.2026.1890675

**Published:** 2026-09-24

**Authors:** Yuanzi Liang, Junqi Hu, Zhiqun Wang

**Affiliations:** 1 Department of Radiology, Aerospace Center Hospital, Beijing, China; 2 Department of Nephrology, Aerospace Center Hospital, Beijing, China

**Keywords:** acute kidney injury, ectopic parathyroid adenoma, hungry bone syndrome, hypercalcemic crisis, primary hyperparathyroidism

## Abstract

A 61-year-old man was admitted to the hospital with fatigue and hypercalcemia, with serum calcium levels as high as 6.16 mmol/L and parathyroid hormone (PTH) levels >5000 pg/mL, accompanied by progressively rising serum creatinine levels (150→266 μmol/L). A neck ultrasound revealed a small nodule in the left parathyroid region, but ^99m^Tc-MIBI imaging identified a focal area of increased radiotracer uptake within the mediastinum. Following multidisciplinary consultation, a thoracoscopic mediastinal mass resection was performed, and pathology confirmed an ectopic parathyroid adenoma (3.2 × 2.8 × 1.5 cm). Postoperatively, PTH levels dropped sharply to 56.17 pg/mL, accompanied by severe hypocalcemia (lowest 1.85 mmol/L) and numbness in the extremities, consistent with the presentation of hungry bone syndrome. Serum calcium levels gradually recovered following intravenous and oral administration of calcium supplements and calcitriol, and renal function also improved significantly. Although all components of this case have been reported individually, the combination of extreme biochemical values, acute kidney injury, and well-documented postoperative course is unusual and instructive. Functional imaging was decisive for localization; postoperatively, hungry bone syndrome should be anticipated and managed proactively. This case demonstrates the full clinical trajectory and offers practical lessons for similar complex presentations.

## Introduction

Primary hyperparathyroidism (PHPT) is one of the most common causes of hypercalcemia, with approximately 85%–90% of cases caused by a single parathyroid adenoma (1). Ectopic parathyroid adenomas account for approximately 20% of PHPT cases, of which only 1%–2% are located in the mediastinum (2). Due to their deep location, mediastinal ectopic parathyroid adenomas are often missed during routine neck exploration, frequently leading to delayed diagnosis and severe complications (2, 3). Video-assisted thoracoscopic surgery (VATS) has become the procedure of choice for mediastinal ectopic adenomas due to its minimally invasive nature and rapid recovery (4, 5). We report a case of a patient who developed a hypercalcemic crisis and acute kidney injury due to a mediastinal ectopic parathyroid adenoma and subsequently presented with classic “hungry bone syndrome” following VATS resection. The case is notable for the extreme degree of PTH and calcium elevation, associated acute kidney injury, and a fully documented postoperative course of hungry bone syndrome, illustrating the value of functional imaging and multidisciplinary management.

## Case report

A 61-year-old male patient was admitted to the hospital presenting with “fatigue for 1 week and elevated serum calcium levels detected 3 days ago”. The patient had developed fatigue 1 week prior without any apparent precipitating factor. He denied hematuria, foamy urine, anorexia, or nausea and vomiting. Three days ago, at a local hospital, his serum calcium was measured at 6.16 mmol/L and his parathyroid hormone (PTH) at 2689.32 pg/mL. Ultrasound revealed a solid nodule in the left parathyroid gland (7 × 4 mm), and chest CT showed a nodule in the anterior mediastinum (2.4 × 3.1 cm). Serum creatinine rose from 150 *μ* mol/L to 250 *μ* mol/L. The patient received fluid resuscitation, salmon calcitonin, and zoledronic acid at the local hospital; upon follow-up, serum calcium was 3.74 mmol/L, and the patient was transferred to our hospital. The patient has a history of hypertension for over 10 years and a history of appendectomy; no other chronic diseases or family history were reported. On physical examination, the temperature was 36.5°C, pulse 72 bpm, respiratory rate 20/min, and blood pressure 117/87 mmHg. The patient was alert and oriented.No palpable masses were detected in the neck, and no significant abnormalities were noted in the heart, lungs, abdomen, or extremities. **Table 1** provides detailed laboratory results.

**Table 1:** Admission laboratory findings.

Parameter	Value	Reference range
Serum calcium (mmol/L)	3.03	2.11–2.52
Serum phosphorus (mmol/L)	0.50	0.85–1.51
Serum magnesium (mmol/L)	0.40	0.75–1.02
Serum creatinine (μmol/L)	258.4	57–111
Blood urea nitrogen (mmol/L)	14.8	3.1–8.0
24-hour urine protein (g/24h)	1.044	<0.15
Parathyroid hormone (pg/mL)	>5000	15–65
Alkaline phosphatase (U/L)	103.7	45–125
25-hydroxyvitamin D (nmol/L)	65.21	>50
Albumin(g/L)	33.7	40–55
Hemoglobin (g/L)	103	130–175
White blood cell count (×10^9^/L)	2.32	3.5–9.5
Platelet count (×10^9^/L)	103	125–350
B-type natriuretic peptide (pg/mL)	607	<100
High-sensitivity troponin I (pg/mL)	16.5	<26.2
ANA	Negative	Negative
ANCA	Negative	Negative

Neck ultrasound: A 7 × 4 mm hypoechoic nodule with a regular shape and internal blood flow signals was observed in the left parathyroid region, adjacent to the dorsal aspect of the thyroid gland. ^99m^Tc-MIBI parathyroid scintigraphy (**Figure 1**): The thyroid demonstrated slightly decreased radiotracer uptake; there is a possibility of an ectopic parathyroid gland in the mediastinum. Chest CT (**Figure 2**): Anterior mediastinal mass.Chest MRI (**Figure 3**): A cystic lesion in the anterior mediastinumis likely.

**Figure 1:**
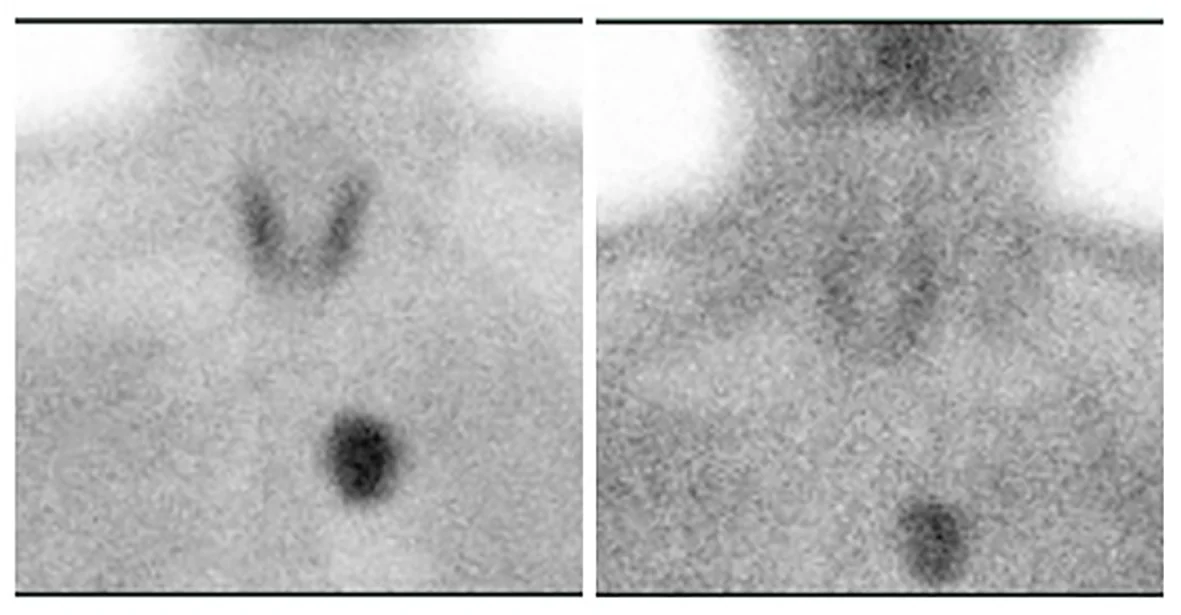
At the 30-minute scan, the thyroid showed slightly decreased radiotracer uptake, and a distinct area of radioactive uptake is visible in the mediastinal region. At the 2-hour scan, radioactivity in the thyroid has diminished, while the mediastinal area of uptake still shows radioactive distribution, consistent with the typical presentation of an ectopic parathyroid adenoma.

**Figure 2:**
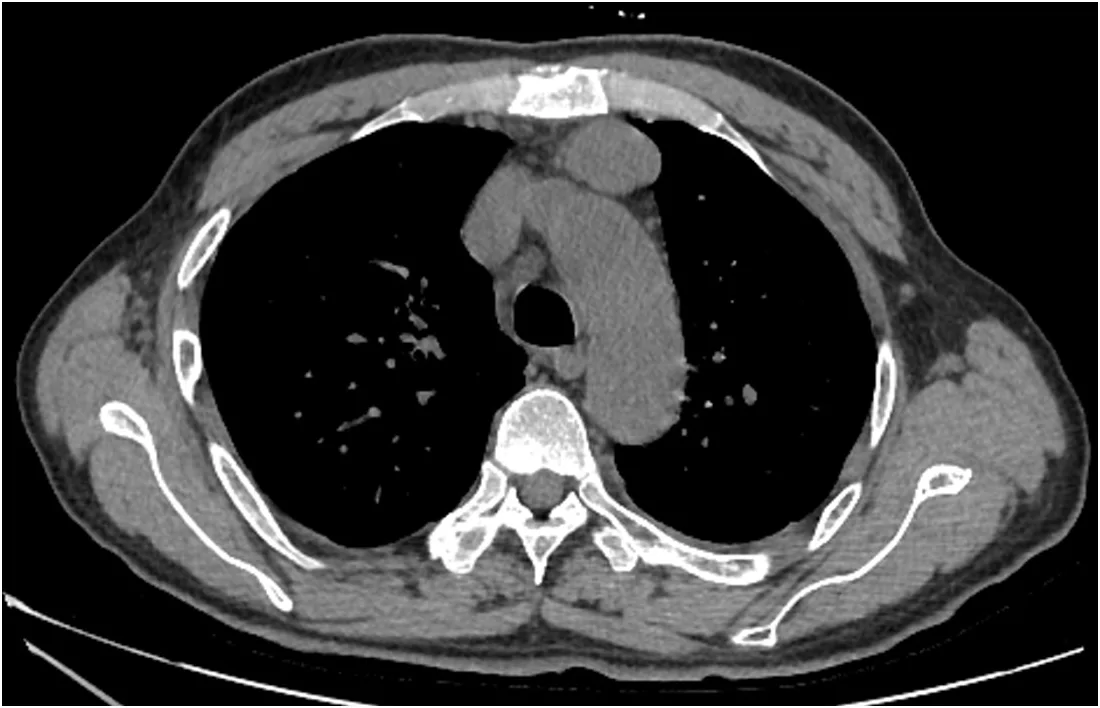
A round, slightly hyperdense lesion in the anterior mediastinum with a CT value of approximately 24 HU.

**Figure 3:**
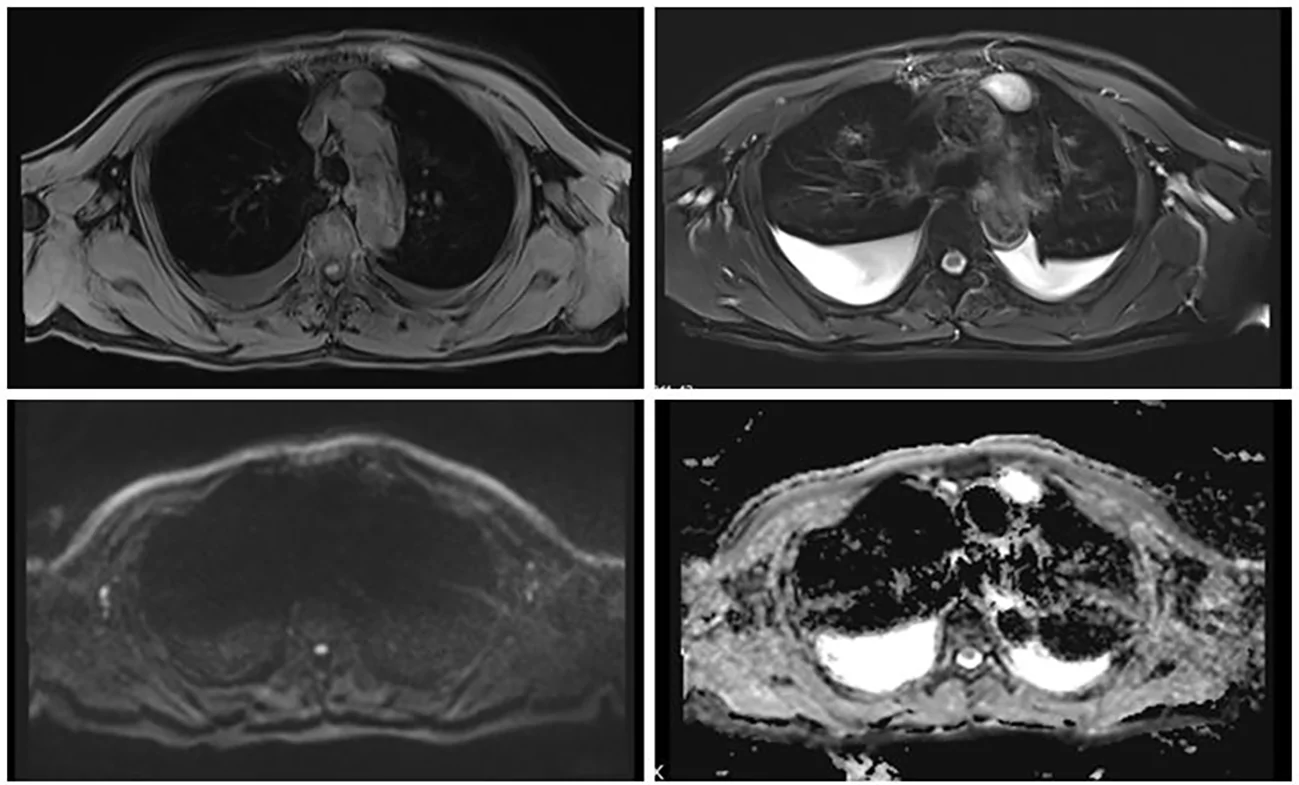
A nodular area of abnormal signal intensity is visible in the anterior mediastinum, measuring approximately 3.1 × 2.3 cm. It appears hypointense on T1-weighted images and hyperintense on T2-weighted images. No signal of restricted diffusion is observed on DWI, and no signal reduction is observed on ADC.

Upon admission, the patient received aggressive fluid resuscitation, diuretics, and salmon calcitonin to lower serum calcium levels. Following treatment, serum calcium levels decreased to 2.22 mmol/L by day 4 after admission, while serum creatinine fluctuated between 237 and 266 μmol/L. Based on parathyroid scintigraphy and CT findings, an ectopic parathyroid adenoma within the mediastinum was considered highly likely. Following a multidisciplinary discussion, the patient underwent “thoracoscopic mediastinal mass resection with pleural adhesiolysis” under general anesthesia 10 days after admission. Intraoperative exploration revealed a firm mass located in the left anterosuperior mediastinum, measuring approximately 3 cm in size, which was completely resected along with the surrounding adipose tissue. Intraoperative frozen section examination was consistent with an ectopic parathyroid adenoma. Immediately after surgery, intravenous calcium gluconate (10%, 100 ml daily) was started. On postoperative day 1, oral calcitriol (0.5 *μ*g twice daily) and calcium carbonate (2.25 g three times daily) were added. Four hours postoperatively, PTH was 56.17 pg/mL and serum calcium was 2.11 mmol/L. On the second day postoperatively, the patient developed numbness around the mouth and in the limbs. Serum calcium levels dropped to 1.85 mmol/L, serum phosphorus to 0.50 mmol/L, and serum magnesium to 0.46 mmol/L, consistent with hungry bone syndrome. Intravenous calcium gluconate was increased to 200 ml daily, and oral calcium carbonate to 3 g three times daily. Serial postoperative serum calcium, phosphorus, magnesium, and PTH levels are shown in **Table 2**. Hypocalcemia (<2.1 mmol/L) persisted for approximately five days and resolved gradually with continued supplementation. On postoperative day 12, intravenous calcium was discontinued, and the patient was maintained on oral calcitriol (0.5 *μ*g twice daily) and calcium carbonate (2.25 g three times daily). Routine postoperative paraffin-embedded histopathology report: (mediastinal mass) ectopic parathyroid adenoma, measuring 3.2 × 2.8 × 1.5 cm; atrophic thymic tissue was observed within the surrounding adipose tissue. Immunohistochemistry: CK(+), CgA (scattered individual +), Syn(-), Ki-67(+<1%), TG(-). On the 12th day postoperatively, serum calcium levels were stable at 2.0 mmol/L or higher and creatinine had decreased to 196.8 *μ*mol/L. The patient was discharged with instructions to continue taking oral calcium supplements and calcitriol, and to undergo regular follow-up tests for electrolytes, renal function, and PTH.

**Table 2:** Serial postoperative serum biochemistry.

Postoperative day	Calcium (mmol/L)	Magnesium (mmol/L)	Phosphorus (mmol/L)	PTH (pg/mL)
POD 1	2.11	0.49	1.15	56.17
POD 2	1.85	0.46	0.5	—
POD 3	1.95	0.42	1.30	—
POD 4	1.79	0.41	1.27	83.88
POD 5	2.03	0.42	1.07	74.98
POD 6	1.92	0.69	1.03	143.84
POD 7	2.12	0.83	0.92	59.80
POD 8	—	—	—	—
POD 9	2.28	0.90	0.77	—
POD 10	2.29	0.62	0.81	—
POD 11	2.25	1.03	0.91	26.67
POD 12	2.32	0.68	0.87	—
POD 13	2.28	0.59	0.79	—
POD 14	—	—	—	—
POD 15	2.18	0.58	0.69	48.79

## Discussion

This case is distinguished by the extreme biochemical derangements (serum calcium 6.16 mmol/L, PTH >5000 pg/mL), the presence of acute kidney injury, and the complete documentation of postoperative hungry bone syndrome. It also illustrates the diagnostic challenge posed by a misleading cervical nodule and the decisive role of functional imaging.

Hypercalcemic crisis, generally defined as serum calcium >3.5 mmol/L, is a life-threatening emergency. Standard management includes aggressive volume repletion, calcitonin for rapid calcium reduction, and subsequent bisphosphonates for sustained control (6). Our patient received this standard regimen, with a prompt decline in serum calcium. The associated acute kidney injury likely resulted from hypercalcemia-induced renal vasoconstriction, impaired medullary sodium transport, and antagonism of antidiuretic hormone (6). In our patient, creatinine fell from 258.4 to 196.8 *μ*mol/L after calcium normalization, suggesting a reversible component, although incomplete recovery indicates possible chronic damage from prolonged hypercalcemia or hypertension.

Given the extremely high calcium and PTH levels, parathyroid carcinoma was considered. However, several features favored benign adenoma: the lesion was well-circumscribed without local invasion or lymphadenopathy; intraoperatively it was mobile and non-fibrotic; and the immunohistochemistry showed very low proliferative activity (Ki-67 <1%). Furthermore, postoperative PTH fell rapidly to normal, which would be unusual for carcinoma (7). Tertiary hyperparathyroidism was excluded by the absence of long-standing end-stage kidney disease or dialysis. Regarding medical therapy, cinacalcet was not used because the patient had an acute hypercalcemic crisis requiring rapid correction and a resectable lesion; cinacalcet is typically reserved for patients who are not surgical candidates or as a bridging therapy.

Hungry bone syndrome is characterized by severe and prolonged hypocalcemia after parathyroidectomy, accompanied by hypophosphatemia and hypomagnesemia (8). Its pathogenesis involves the abrupt cessation of PTH-driven bone resorption while osteoblastic activity continues, leading to rapid mineral deposition into demineralized bone. High-risk features include very high preoperative PTH, high alkaline phosphatase, large adenoma, and vitamin D deficiency. In our patient, PTH was >5000 pg/mL and the adenoma was large; ALP (103.7 U/L) and 25-hydroxyvitamin D normal (65.21 nmol/L). Despite immediate postoperative calcium infusion, serum calcium fell to 1.85 mmol/L and remained below 2.1 mmol/L for five days, satisfying diagnostic criteria for HBS. Treatment with high-dose calcium and calcitriol eventually restored normocalcemia. This course suggests that in patients with extreme preoperative PTH, even more aggressive prophylactic supplementation from the first postoperative hours may be warranted.

Traditional sternotomy or thoracotomy carries significant morbidity. VATS offers excellent exposure with minimal trauma, less blood loss, and shorter hospital stay (4, 5). In a 23-year single-center study, Liu et al. found VATS superior to open surgery in operative time and blood loss (4). Chavez et al. confirmed its safety and cost-effectiveness (9). Our case adds to this evidence.

The patient was initially managed by nephrology, with nuclear medicine providing precise functional localization, thoracic surgery performing VATS, and subsequent careful management of hypocalcemia. This model is essential for rare ectopic lesions.

## Limitations

In this case, preoperative bone density testing and whole-body bone scintigraphy were not performed, making it impossible to quantify the extent of bone loss. And 24-hour urinary calcium excretion were not measured during the patient’s hospitalization. The postoperative follow-up period has been relatively short, and the long-term outcomes regarding renal function and PTH levels require further observation.

## Conclusion

This case of mediastinal ectopic parathyroid adenoma presented with hypercalcemic crisis, acute kidney injury, and postoperative hungry bone syndrome. Functional imaging was essential for localization, and VATS provided definitive cure. Proactive management of hungry bone syndrome is critical in high-risk patients. The case offers practical insights for clinicians facing similar complex presentations.
